# Cognitive Penetration and Attention

**DOI:** 10.3389/fpsyg.2017.00221

**Published:** 2017-02-22

**Authors:** Steven Gross

**Affiliations:** Department of Philosophy, Johns Hopkins UniversityBaltimore, MD, USA

**Keywords:** cognitive penetration, attention, perception, top–down, expectation

## Abstract

Zenon Pylyshyn argues that cognitively driven attentional effects do not amount to cognitive penetration of early vision because such effects occur either before or after early vision. Critics object that in fact such effects occur at all levels of perceptual processing. We argue that Pylyshyn’s claim is correct—but not for the reason he emphasizes. Even if his critics are correct that attentional effects are not external to early vision, these effects do not satisfy Pylyshyn’s requirements that the effects be direct and exhibit semantic coherence. In addition, we distinguish our defense from those found in recent work by Raftopoulos and by Firestone and Scholl, argue that attention should not be assimilated to expectation, and discuss alternative characterizations of cognitive penetrability, advocating a kind of pluralism.

## Introduction

What we think can affect what we see. For example, if you want some chocolate and think it is to your left, you might turn to look that way. What you now see will differ from what you saw before: a half-eaten bar on the counter rather than the empty cupboard. In this case, a kind of attention plays a mediating role: what you think causes you to change the orientation of your gaze, which in turn has obvious effects on what you see.

What does this show about the relation between cognition (that is, higher cognition, or conception) and perception? Certainly, it shows that there are ways the former can causally affect the latter. Does it show something more significant? Might it require a reconception of cognitive architecture—perhaps even call into question the distinction between perception and conception? Might it deprive us of a theory-neutral basis for adjudicating among competing hypotheses, or undermine perception’s apparent role in providing independent warrant to beliefs?

At least for the case at hand, this seems unlikely (however, interesting it may otherwise be). Because cognitive effects on perception via bodily movement are both unsurprising and indirect, it is unclear how they might challenge or reshape the distinction between seeing and thinking. Because this sort of attention can be so readily redirected, it is not obvious how it might render potential evidence inaccessible when comparing theories. And because it seems to filter information (for example, having you look *here*, not *there*) but not alter that which it selects, it would seem only to constrain the basis for one’s beliefs, not to affect the epistemic relevance of what one *does* see.

But the case at hand is particularly unsubtle. In other cases, one’s eyes can move in perceptually consequential ways without one’s realizing it, even upon reflection: eye-tracking was required to demonstrate the role of saccades in flipping ambiguous images (e.g., Stark and Ellis, 1981). Moreover, attention is not limited to *overt* attention (the reorientation of gaze through bodily movement). Even with one’s gaze fixed, *covert* attention can shift among locations, features, and objects. In such cases, the implications for perception’s epistemic function and the perception-cognition relation are less clear and more controversial. One example: when attentional effects on perception are less obvious, so are their potential biasing effects on belief—whether malign (the neglect of contrary evidence in confirmation bias) or beneficial (when attention prevents us from missing what is most relevant). Another example, which will play a larger role in our discussion: because the mechanisms of covert attention seem more bound up with perceptual processing itself, a cognitive influence upon them appears to amount to a *direct* effect on perception in a way that cognition’s effects upon overt attention do not.

Questions concerning cognition’s bearing on perception are often framed in terms of the cognitive penetrability or impenetrability of perception. Applied to our topic, the question is thus whether cognitively driven *attentional* effects on perception can amount to cognitive penetration. But talk of cognitive penetration gets cashed out in various ways, so that the answer depends on just what cognitive penetration is supposed to be.

In what follows, we approach the question using Zenon Pylyshyn’s characterization of cognitive penetration. It was he who coined the term, but, more importantly, his conception is well-motivated, as we will indicate in a moment. A further justification for our focus is that, although much subsequent discussion has centered on Pylyshyn’s claims, his resources for precluding attentional effects from the purview of cognitive penetration have not been fully explored or exploited either by Pylyshyn himself or his other defenders.

Pylyshyn’s concern is the degree to which visual perception is “continuous” with cognition. More specifically, he asks whether early visual states interact with cognitive states in the way cognitive states do with one another—in particular, by mirroring rational relations. Early vision would be cognitively penetrated, on his view, if “the function early vision computes is sensitive, in a semantically coherent way, to the organism’s goals and beliefs” (Pylyshyn, 1999a, p. 343). With this characterization in hand, he argues that cognitively driven attentional effects, though they provide the primary means by which cognition affects perception, do not amount to cognitive penetration. Indeed, showing that various phenomena offered in evidence of cognitive penetration in fact involve subtle attentional effects is among his principle strategies for rebutting others’ claims. Thus, he counters Churchland’s (1988) discussion of cognitive effects on the perception of ambiguous figures by adducing the evidence mentioned above for the role of eye movement in bringing about perceptual flips.

We agree that, when cognitive penetration is understood in Pylyshyn’s way, cognitively driven attentional effects on perception do not amount to cognitive penetration—but not for the reason Pylyshyn emphasizes. Pylyshyn maintains that attentional effects occur either before or after early vision and thus do not directly affect early vision itself. Critics have focused on this claim, replying that in fact attention is bound up with perceptual processes at all levels. This in part accounts for a rising tide of attention-based cognitive penetrability claims (e.g., Lupyan, 2015; Mole, 2015; Wu, forthcoming). But there are other bases for excluding attentional effects from the purview of cognitive penetration. In particular, cognitively driven attentional effects fail to satisfy the requirement that there be semantically coherent sensitivity to cognitive states—or so we shall argue. Along the way, we differentiate our defense from those found in recent work by Raftopoulos (2009) and Firestone and Scholl (2015, 2016); and we respond to views that would assimilate attention to expectation and thereby argue that Pylyshyn’s criterion can be met.

But are the significant questions best framed in Pylyshyn’s terms? We conclude by considering other conceptions of cognitive penetration advanced in the literature, some of which do and some of which do not count cognitively driven attentional effects on perception as cognitive penetration. We consider as well how one might decide among them. In the end, we advocate a kind of pluralism, suggesting that there may be no one question of cognitive penetrability, but a variety of interesting successors—and so no one answer to the question concerning attention with which we begin. Pylyshyn’s conception is motivated, but others may be as well. Of course understanding the various ways cognition and perception interact and their upshot is more important than determining if there are phenomena worthy of Pylyshyn’s label. But this conclusion does not undermine the interest of our earlier exploration of attention and cognitive penetration as *Pylyshyn* defines it: first, it is *among* the various interesting questions; and, second, considering questions of cognitive penetrability, including what cognitive penetrability should be, is a useful strategy for *delineating* the various interesting questions, even if it is a ladder one then throws away.

## Attention and Cognitive Penetration in Pylyshyn’s Sense

To see why Pylyshyn holds that cognitively driven attentional effects on perception do not amount to cognitive penetration (and how else it might be defended), we should first clarify his conception of cognitive penetration. A few remarks concerning the relevant kind of attention will also prove useful.

### Pylyshyn’s Conception of Cognitive Penetration

Roughly, cognition penetrates perception just in case it causally affects perception in the right kind of way (that is, subject to some sort of further constraints on the kind of causal effect). But views vary as to what counts as cognition, perception, and causing in the right kind of way. These differences matter for whether attentional effects can count as cognitive penetration. Pylyshyn, we saw, is concerned with whether “the function early vision computes is sensitive, in a semantically coherent way, to the organism’s goals and beliefs.” As he also puts it, cognitive penetration requires that early vision “can be altered in a way that bears some logical relation to what the person knows;” an instance of cognitive penetration must “alter the contents of perceptions in a way that is logically connected to the contents of beliefs, expectations, values, and so on” (Pylyshyn, 1999a, p. 343). The relevant cognitive states—the admissible *source* of would-be cognitive penetration—thus comprise for Pylyshyn the so-called propositional attitudes. The relevant *target* of his impenetrability claim is not perception *tout court*, which he claims *is* cognitively penetrable (Pylyshyn, 1999a, p. 344), but just so-called early vision, a substantial portion of the perceptual processes implicated in visual perception. It is question just what early vision comprises. Pylyshyn mentions, for example, the calculation of stereo, motion, size, and lightness constancies (Pylyshyn, 1999a, p. 344). But we need not pursue the matter, since the considerations we ultimately adduce in Pylyshyn’s defense do not rest on a particular conception of, and are not limited to potential effects on, early vision.

Finally, for the causal effect of cognition on early vision to count as the “right kind,” the representational contents of the cognitive states and of the affected early vision states must be related in a way that satisfies two conditions. First, early vision must itself have “access” (Pylyshyn, 1999a, p. 344 and *passim*) to the cognitive states. The cognitive states must exert their influence because early vision’s computations take their contents into account by operating over them, not just because the cognitive states have effects on *other* states over which early vision computes. In this sense, the influence must be *direct*. Second, the contents of the cognitive states and the contents of the affected early vision states must stand in a relation of semantic coherence—or, as he also puts it, a logical or rational relation:

We sometimes use the term “rational” in speaking of cognitive processes or cognitive influences. This term is meant to indicate that in characterizing such processes we need to refer to what the beliefs are about—to their semantics. The paradigm case of such a process is inference, where the semantic property truth is preserved. But we also count various heuristic reasoning and decision-making strategies (e.g., satisficing, approximating, or even guessing) as rational because, however, suboptimal they may be by some normative criterion, they do not transform representations in a semantically arbitrary way: they are in some sense at least quasi-logical. This is the essence of what we mean by cognitive penetration: it is an influence that is coherent or quasi-rational when the meaning of the representation is taken into account (Pylyshyn, 1999a, p. 365, fn. 3).

These formulations raise further questions, but the basic idea of one representation not just causing another, but providing a reason for it, will suffice for our purposes.^1^ The requirements of directness and semantic coherence articulate the kind of “sensitivity” cognitive penetration requires. It is not enough that the contents of early vision states be sensitive to those of cognitive states in the weaker sense of depending counterfactually, statistically, or in a law-like manner upon them. They must also do so in virtue of early vision itself operating over the cognitive states in a manner that mirrors a rational relation.

Not all parties to cognitive penetrability disputes characterize the would-be phenomenon in this way. In particular, some drop the requirement of directness, and many drop the requirement of semantic coherence. We canvass some of these alternatives below. For now, we underscore Pylyshyn’s motivation. Pylyshyn is interested in whether vision and cognition are “continuous,” as New Look psychology suggests (Bruner, 1957). A central feature of propositional attitudes is that they *do* directly affect one another in semantically coherent ways. Indeed, their availability for rational inference about what to believe and what to do—and the conceptual structure this imposes upon them—is among their most important functional features. If perceptual states—more specifically, states of early vision—interacted with propositional attitudes in a similar way, this would be a strong argument for a crucial continuity with them. If they do not, it is a crucial *dis*continuity. Establishing Pylyshyn’s thesis thus helps mark and preserve at least an aspect of the perception-cognition distinction itself. It is important to note, however, that the (not necessarily exhaustive) distinction need not rest solely upon this discontinuity. For example, one might also differentiate perceptions and cognitions by their relative stimulus-dependence—more specifically, whether it is their function to represent the here and now (cf. Pylyshyn, 1999a, p. 343; also Burge, 2010). (Pylyshyn, 2002, however, rejects one oft-proposed basis for drawing a perception/conception distinction: that the former have iconic and the latter symbolic representational formats.)

### Kinds of Attention

Attention comprises a variety of phenomena and is perhaps something of a motley (Allport, 1993). But we can pinpoint, or at least minimally clarify, what kind of attentional effect is at issue here.

Attentional phenomena sub-divide in various, sometimes cross-cutting ways. Attention can be external (selecting and modulating sensory information) or internal (selecting, modulating, and maintaining memories, choices, responses, and other non-sensory representations) (Chun et al., 2011). Our focus is of course external attention, since we are considering a candidate case of the cognitive penetrability of *perception*. As mentioned, if external attention involves the movement of sensory organs, it is overt; otherwise, it is covert. It is widely agreed that cognitive effects on perception mediated by overt attention—as with our example of looking to the left because one wants some chocolate and believes that is where it is—should not count as cognitive penetration, because the effect is not direct or because admitting them would render the topic uncontroversial. Thus, our focus is covert attention. Note, though, that cases of pure overt attention shifts may be atypical. For among the hypothesized functions of covert attention is to prepare or guide overt attention shifts, for example by highlighting a target for eye movement or visual search (Kowler, 2011; Nakayama and Martini, 2011). (Another hypothesized function, relevant to social cognition, is to allow undetectable attention shifts—see Laidlaw et al., 2016) Cases of cognitively driven overt attention shifts could therefore involve cognitive penetration—albeit not in virtue of their overt aspect—if concomitant cognitively driven covert attentional shifts can amount to cognitive penetration.

Cases of covert attention can be classified by what drives them. Exogenous attention is driven directly by external cues in a bottom–up fashion, as when attention is captured by a sudden noise. Endogenous attention is driven from within in a top-down fashion. The top–down processes involved in endogenous attention can, however, occur in response to an external cue, as is typically the case in experimental settings. Endogenous and exogenous cues differ according to whether they must be in some sense understood. For example, whereas an exogenous cue might increase attention to a location by simply occurring there, an endogenous cue might do so by indicating that location via an arrow or by a symbolic description (‘up’). Because endogenous cues must be understood, they achieve their attentional effects by engaging mechanisms and cortical areas different from those required for exogenously driven attentional effects; and there is a corresponding difference in time-course: 300 ms from endogenous cue to attention shift, compared to a 100–120 ms peak for exogenous cues (Carrasco, 2011). However, that a cue generates an endogenous shift in attention might not of itself entail that the shift is cognitively driven in Pylyshyn’s sense. It is possible that the relevant representation or association (e.g., of an arrow and a direction) is contained within perception itself, or comes to be over a course of trials (Pratt and Hommel, 2003; Stevens et al., 2008; Pratt et al., 2010). This is just a particular instance of a point proponents of cognitive *im*penetrability have always emphasized: that top–down does not entail cognitive, since top-down processing can occur within perception itself (Fodor, 1983; Pylyshyn, 1999a). That said, cognitively driven attention is of necessity endogenous. So, we will only be concerned with it.

Raftopoulos and Lupyan (2017), in laying out the research topic to which this paper is a contribution, make special mention of pre-stimulus cues. It is thus worth noting that the only role external cues play in endogenous attention is to generate the internal states that then cause the attention shift. In that sense, the cue’s role is indirect and essentially irrelevant once its job is done. Perhaps, then, there is no *special* question of whether cognitively driven attentional effects on perception *brought on by pre-stimulus cueing* (as opposed to, say, an unprompted decision to attend in a certain way) count as cognitive penetration—that is, nothing further to ask beyond whether cognitively driven attentional effects on perception count as cognitive penetration more generally. This turns out to be the case given Pylyshyn’s conception of cognitive penetration: what brought about the cognitively driven attentional effect will be irrelevant to the considerations we adduce. (There may be, however, as indicated above, a question whether particular endogenous attentional effects are in fact cognitively driven.)^2^

Finally, cases of covert attention can also be classified by their object—i.e., what one attends to. Work over the last few decades typically distinguishes spatial, feature-based, and object-based attention (Carrasco, 2011). (For simplicity, we bracket temporal attention, a special case that is arguably not as well understood and perhaps spans the external/internal divide. See Nobre and Coull, 2010; Phillips, 2012; Gross, in preparation). This classification matters for certain arguments mentioned below, but its importance will fade once we focus on our alternative defense of Pylyshyn’s claim.

## Pylyshyn’s Argument and its Critics

Pylyshyn maintains that cognitively driven attentional effects—whether overt or covert—do not provide examples of cognitive penetration of early vision. His main argument is that attentional effects either help determine the inputs to early vision or help select from among its outputs.^3^ Attentional effects may *in*directly affect early vision (even selection effects *after* early vision may indirectly affect early vision—for example by causing an effect on its subsequent inputs). But because they involve no *direct* effect on early visual processing itself, they do not exhibit a way “the function early vision computes is sensitive, in a semantically coherent way, to the organism’s goals and beliefs.”

Critics respond that attentional effects are found at all levels, or stages, of processing. Thus, either they occur in early vision or, if early vision is to be insulated from them, there seems nothing substantial left for early vision to be. Talk of levels can be cashed out in various ways. Yeh and Chen (1999), for example, argue that results finding attentional effects at various *cortical* levels leave little space for an attentionally insulated stage of early vision. (Cf. Lupyan, 2015, p. 560: “As is now well-known, attention modulates processing at all levels of the visual hierarchy …. *Prima facie*, these findings appear to be devastating for opponents of the [cognitive penetrability of perception] thesis ….”) Pylyshyn (1999b, p. 410) replies that one cannot assume a straightforward mapping between cortical and *computational* stages, and it is the latter with which he is concerned. However, subsequent attention research has arguably trended towards a convergence of behavioral and neurophysiological data that shifts the burden onto anyone who would defend Pylyshyn’s claim that attentional effects are external to early vision. As Carrasco (2011, pp. 1485–1486) writes in summing up the preceding 25 years of research:

Initially, there was a great deal of interest in categorizing mechanisms of vision as pre-attentive or attentive [i.e., involving selection after early vision]. The interest in that distinction has waned as many studies have shown that attention actually affects tasks that were once considered pre-attentive, such as contrast discrimination, texture segmentation and acuity…. this review focuses on the effect of attention on basic visual dimensions where the best mechanistic understanding of attention to date has been achieved, such as contrast sensitivity and spatial resolution [… and …] motion processing …. due to the existence of models of these visual dimensions, as well as to the confluence of psychophysical, single-unit recording, neuroimaging studies, and computational models, all indicating that attention modulates early vision.

Note that an “all levels” claim can affirm that the fundamental function of attention is to cull inputs. This is a natural idea whose mechanisms are becoming better understood. (For example, recent work suggests that attention’s primarily function is to select among stimuli and thus reduce the cost of stimulus mixing in cortical response, not to increase response sensitivity or reduce noise via an increase in gain in relevant areas. See Pestilli et al., 2011; Orhan and Ma, 2015). What an ‘all levels’ claim rejects is just that this culling of inputs does not occur *inter alia* within early vision. (Note that the inputs to be culled could be sensory inputs or inputs provided by one computational mechanism to the next.) Firestone and Scholl’s (2016, pp. 23–24) reply—citing, incidentally, the same Carrasco survey—to the objection that not all attentional shifts are like moving one’s eyes would thus cut no ice if directed against the “all levels” complaint:

… fundamentally, “attention is a *selective* process” that modulates “early perceptual *filters*” (Carrasco, 2011, pp. 1485–1486, emphasis added). This is what we mean when we speak of attention as constraining input: attention acts as a “filter” that “selects” the information for downstream visual processing, which may itself be impervious to cognitive influence.

If this selection occurs at “all levels”—in particular, at each stage of processing within early vision—it remains the case that no substantial component of visual perception may remain that is “impervious to cognitive influence.”

There are indeed ways one might attempt to defend Pylyshyn’s claim that attentional effects are external to early vision, either preceding or following it. But their prospects are inessential to our main point: we argue that one can in any event defend on other Pylyshian grounds the claim that cognitively driven attentional effects on early vision do not amount to cognitive penetration. However, because debate has focused on where attentional effects are felt, we provide a brief indication of possible directions one might explore on this front. Theeuwes (2013) argues that all feature-based attention involves bottom-up priming. Having perceived a certain feature, one’s perceptual system is then primed to perceive it again, regardless of its location or the object that has it. Previous work, he argues, missed this by presenting subjects with blocks of trials that did not control for stimulus history. When stimulus history is controlled for, feature-based attentional effects disappear. If he is right, this removes one candidate category of cognitively driven attentional effects. Recent work indicates that object-based attention is likewise subject to the effects of stimulus history (Lee et al., 2012). A rather hopeful defender might speculate that it too might all be bottom-up. Alternatively, she might pin her hopes on the minority view that apparent object-based attentional effects are really spatial (see Reppa et al., 2012 for references and critical discussion). But, failing that, it may be conceded in any event that object-based attentional effects occur only after early vision. This would leave spatial attention. As we noted, the work of Carrasco and others suggests that attentional effects are entwined with perceptual processing throughout early vision. But Schneider (2006, 2011) attempts to explain their results by positing an attentional effect on salience and a post-perceptual decision bias in favor of salient items, rather than an attentional effect on perceptual content. On this view, salience, though a property of perceptions, is not something itself represented in perception. By affecting salience, attention might have a *kind* of effect on perceptual processing at “all levels” on such a view, but not the kind relevant to cognitive penetration—viz., an effect on content. It would only have that kind of effect post-perceptually, at the level of perception-based judgment. (See Beck and Schneider, forthcoming for philosophical discussion, replying to Block, 2010.) If these moves (or others) were to pan out, they would vindicate Pylyshyn’s claim that attentional effects—or at least the relevant ones—are all external to early vision. Many will consider that a big *if*. We now argue that the claim it was intended to subserve—that cognitively driven attentional effects on early vision do not amount to cognitive penetration—can be defended in any event. Discussion need not fixate on the locus of attentional effect.

## An Alternative Defense of Pylyshyn’s Claim

If cognitively driven attentional effects were all external to early vision, that would suffice to show that they are indirect: the function early vision computes would not be sensitive to them in a semantically coherent way. But showing that they are in fact internal does not yet show that Pylyshyn’s requirements on cognitive penetrability are satisfied. Are there grounds for thinking they are not?

It might be thought that it does not matter where in processing the effect is felt: the effect must be indirect simply because it is mediated by attention, so that, even when cognitively driven attention exerts its influence on early vision (not just on inputs to early vision), this involves *first* cognition affecting attention, which then *in turn* affects early vision. But Mole (2015) argues that whatever plausibility this thought may have for overt attention, it requires a mistaken picture of covert attention as a faculty or capacity distinct from perception and capable of causally affecting it—as opposed to its just being itself a certain kind of effect in perception.^4^ If covert attentional effects are a part of perceptual processing, then, pending the identification of other mediating factors, cognitive effects on perception supposedly mediated by covert attention *are* direct effects on perception.

The indirectness objection, however, can be pressed in a more subtle way. To see how, consider what occurs when cognitively driven attention affects early vision (not via an effect on sensory input to early vision). The decision to attend can spring wholly from within (we consider such a case below), but in a typical experiment the subject responds to an endogenous cue—what is in effect an instruction from the experimenter. For example, if the cue is an arrow pointing up or the word ‘up,’ it is an instruction to attend *there*. If the case is to satisfy Pylyshyn’s criteria for being *cognitively* driven, the subject will come to have an intention or other action-directed attitude to attend there. She will do this on the basis of such other attitudes as her belief that she has been instructed to attend there, her desire to cooperate, etc. Our suggestion is that these attitudes generate what we might call an *attentional command* to attend there, which—on a causal-computational account—would exert its influence on perceptual processing, affecting perceptual content (at least on the common view suggested by Carrasco’s work and others’—we put aside here Schneider’s animadversions). If the ascription of this attentional command seems fanciful, consider that it is common for computational models of perceptual attention to include attentional parameters that weight the effect of sensory signals (e.g., Lee and Maunsell’s (2009) divisive normalization model, brought to bear on cognitive penetration in Wu (forthcoming)). ‘Attend to this, this much’ is a natural way to gloss their content. And possess content they must if we are so much as to have a candidate case of cognitive penetrability in Pylyshyn’s sense. (In discussing attention and expectation below, we consider models that would dispense with such parameters.)^5^

Now, if the directness aspect of Pylyshyn’s criterion is to be satisfied, perception must have access to and operate over relevant cognitive states: the values of cognitive states must be among the inputs to perceptual processes. One way to defend Pylyshyn’s cognitive impenetrability claim, then, is to argue that (1) the attentional command is not itself a cognitive representation, and (2), although the attentional command plays a role in perceptual processing, the attitudes that generate it do not. The attitudes that generate the attentional command are thus not accessed, and the attentional command is not cognitive; so, no cognitive state is accessed. Perception, by not accessing the cognitive states themselves, would thus not be in that sense sensitive to them; it would only feel the causal effect of those states. We could still say, with Mole (2015), that there is a sense in which cognition affects perceptual processing in an unmediated way. For the cognitive states, on this view, could directly generate the attentional command, itself a part of perceptual processing. And yet, in another sense, we would have to say that there is mediation after all: for, though the attentional command may be directly generated by cognitive states, the attentional *effect* on perception would not be. We can think of the effect either as the result brought about or as the bringing about of the result. In the latter sense, the attentional effect consists in the attentional command exerting its influence and thereby affecting perceptual content (the actual transition from one representational state to another, as brought about in part by the attentional command—in other words, the calculation of the function in which the attentional command is a term). In the former sense, it is just the resulting perceptual state (or perhaps some aspect it would otherwise not have had). Either way, the attentional effect would not be directly generated by cognitive states. Moreover, consider the state that *is* directly affected—the attentional command. Though it is perhaps (if we deny it cognitive status) a representational state *in* perception, it is not itself a perceptual state, at least in the sense of a state whose function is to represent the here and now. Thus, cognition’s direct effect on it does not constitute cognitive penetration of perception.

Perhaps, when all is said and done, this is correct. But it might not be the most convincing way to defend Pylyshyn’s position. For it is unclear on what basis one can compellingly persuade a proponent of cognitive penetrability that the attentional command is not a cognitive representation. In particular, to base one’s case on the fact that it interacts with perceptual representations would just beg the question: that perceptual processes can have access to cognitive states is precisely what a proponent of cognitive penetration in Pylyshyn’s sense claims.

A stronger argument instead incorporates the preceding considerations into a dilemma. For if the attentional command *is* considered a cognitive state, its influence on perception runs afoul of the semantic coherence constraint—so that cognitive penetrability is blocked whichever status one assigns the attentional command. Recall that the semantic coherence constraint requires that the content of the accessed cognitive state bear an inferential relation to the content of the resulting perceptual state. Attending may exert a causal effect on what one sees. But it does not provide an epistemic basis for it. Here a comparison with turning on a light is appropriate (cf. Firestone and Scholl, 2015, p. 8). Turning on a light—perhaps in response to a request—might enable one to see that there is something red there, but not because the turning on of the light is evidence for it. Matters stand otherwise in cases where, according to Pylyshyn, the semantic criterion is met. In late vision, he claims, we might draw upon various beliefs to identify some object. If early vision outputs a representation of an object as having such-and-such shape and coloring, etc., we may access any number of beliefs in coming to then represent the object as Ms. Jones—for instance, beliefs about what Ms. Jones, as opposed to other persons and things, looks like (Pylyshyn, 1999a, p. 344). The content of these beliefs do not just cause, but provide an epistemic basis for the resulting representation of *this* as Ms. Jones. Similarly, for the claimed influence of color memories (that hearts are red, bananas yellow) on color perception (Delk and Fillenbaum, 1965; Macpherson, 2012; Gross et al., 2014; Witzel and Hansen, 2015).

Indeed, intentions and commands are not the *sorts* of states that can provide reasons in the relevant sense. They do not provide epistemic grounds, though they can be related to reasons for action. To attend is a kind of act (a *mental* act—cf. O’Brien and Soteriou, 2009); and, unless it is just a whim, when one forms an intention to attend, one does so on the basis of reasons to attend—in the experimental setting, because you are instructed to so attend and want to cooperate. There is thus an appropriate semantic relation between the relevant cognitive attitudes and the attentional command. Arguably, there is also an appropriate semantic relation between the attentional command and the carrying out of what it commands (viz., the mental act of attending itself). But what there is not is a semantic relation between the attentional command (or the attending) and the resulting perceptual state, the state that exhibits the attentional effect. One might have good reason to turn on a light, and one’s doing so might cause you see to see a red thing there. But the reasons for turning on the light (viz., because you were asked) do not supply any epistemic basis for what you see—that is, for there being something red there—nor does your turning on the light constitute any such reasons. Just so with your reason for attending and for the attending itself: they causally affect what you see, but are not themselves grounds for it.

This is not to say that the directness requirement of Pylyshyn’s conception of cognitive penetration plays no role in turning aside challenges arising from covert attentional effects. To see this, consider the following objection. It might be worried that the argument just given hinges on the kind of case we are considering, where one has simply followed the experimenter’s instruction. But perhaps things are otherwise with at least some more internally generated intentions to attend. Suppose one decides to attend on the basis on some belief about what one will see. For example, you need something red to balance out a design and believe something red is over there. Attending there will raise the probability of your getting something red. So, you attend there and, as a result, see something red there. Here we seem to have an appropriate semantic relation between a cognitive state (the belief that something red is there) and the resulting perception (a visual representation as of something red there).

But even though the belief in part causes the perception and their contents seem to stand in an appropriate relation, it is not the case that the perceiver or her visual system *treats* the belief as evidence for what she sees. This point can be developed in terms of directness: the visual system does not itself access, and so does not take into account, the person’s belief that something red is there; it is just influenced by the action command that in part results from the belief, given how one reasoned what to *do*. Moreover, the worries raised above about appealing to directness do not apply here: it is contentious to deny that the action command is a cognitive state, but less so to deny that the belief that helps generate the action command in a case like this is not itself accessed by early vision—at least insofar as the belief’s *attentional* effect is concerned (we return to this caveat in discussing attention and expectation below).

Perhaps one may argue that in fact the semantic coherence constraint, properly understood, is *also* not satisfied in this case. Consider the distinction between one claim’s providing a reason for another and one claim in fact being the reason *for which* someone upholds a claim. (For example, it might be that A entails B, and one believes both A and B, but one does not believe B on the basis of A because one does not realize that A entails B—nor does one, or one’s reasoning capacity, otherwise encode or “embody” the entailment.)^6^ If we may apply an analogous distinction in theorizing about the visual system, then we might suggest that semantic coherence requires not just that the cognitive state in part cause the perception and that as a matter of fact there be a semantic relation between their contents, but that the content of the former be part of the *basis* upon which the content of the latter is generated. This might seem to eliminate the need to advert to directness in replying to such cases after all. But whether this is so depends on what precisely providing a basis requires. A natural cashing out would require directness: being able to access and operate over the cognitive state. If so, this formulation of semantic coherence would simply build in directness. Note though that, even if semantic coherence were construed broadly to include directness, it would not collapse the two constraints: semantic coherence would still go beyond directness. What we have seen is that attentional parameters in visual computations provide an example of how representations can be accessed and operated over without their role in the computation being appropriately inference-like in Pylyshyn’s sense. Otherwise put, they show that, even though a computational transition might itself be deemed an inference, or inference-like, not all elements of the computation need be (quasi-)reason-giving. Attentional weights affect computations in a different way.

Let us take stock. We suggest that, for cognitively driven attentional effects on perception to amount to cognitive penetration, there must be propositional attitudes that generate an attentional command. The attentional command finds a place in computational models of perceptual processing along the following highly schematized lines: *f*(s, *a*) = *p*—where ‘s’ represents the sensory signal, ‘*a*’ the attentional command (attentional weighting), and ‘*p*’ the resulting perceptual state.^7^ This introduces a variety of candidate loci for cognitive penetration, in part depending on whether the attentional command is a cognitive state or not. The candidates are: the attitudes’ effect on the command, the command’s effect on the perception, and the attitudes’ effect on the perception. But the attentional command does not stand in the appropriate semantic relation to the resulting perceptual state for it to penetrate perception. And if one maintains further—albeit contentiously—that the attentional command is not a cognitive state, then it is not even a candidate source of penetration. The relation of the generating attitudes to the attentional command does satisfy the semantic criterion. But this is irrelevant if the attentional command is itself a cognitive state and so not a candidate object of penetration. And if the attentional command is *not* a cognitive state, still, it is not a perception (and thus not a candidate object of cognitive penetration), even if it is a representation in perception in the sense of being operated over in perceptual processing. Finally, though the attitudes that generate the attentional command can sometimes have reason-giving content relative to the resulting perceptual state, in such cases the directness requirement is violated, since perceptual processing, so far as the attentional effect is concerned, does not access—in that sense is not sensitive to—the content. (And this might violate semantic coherence as well on a broad construal.)

Cognitively driven attentional effects on early vision thus do not provide examples of cognitive penetration in Pylyshyn’s sense. But not for the reason he emphasizes. Even if there are attentional effects that do not occur before or after early vision, they still fail to satisfy Pylyshyn’s requirements—either of directness or of semantic coherence.

## Comparison with other Defenses

We can sharpen these points by differentiating our defense from Raftopoulos’s (2009) and Firestone and Scholl’s (2015, 2016) defenses of similar positions.

### Raftopoulos

Raftopoulos (2009) argues that perception—by which he means early vision *sans* sensation—is not cognitively penetrated. Drawing in part upon Lamme and colleagues (e.g., Lamme, 2003), Raftopoulos argues that early visual processing culminates around 120 ms after stimulus onset, following a feed-forward sweep that leads to the establishment of locally recurring networks; post-perceptual cognitive processes involve later top-down feedback from higher cortical areas. His emphasis on time-course provides one way of supporting Pylyshyn’s suggestion that computational stages do not necessarily line-up with location in the cortical hierarchy, since later temporal stages reuse areas implicated in earlier stages. But, *contra* Pylyshyn on attention, Raftopoulos adduces evidence that within this time-frame, one finds cognitively driven attentional effects, stemming from pre-stimulus cueing, upon early vision.

Raftopoulos argues that, nonetheless, these attentional effects do not constitute cognitive penetration. For, though they facilitate processing, they do not affect the resulting perceptual content (e.g., Raftopoulos, 2009, p. 83). This claim seems in tension with his later suggestion that such attentional effects constrain the interpretation of ambiguous figures (Raftopoulos, 2009, pp. 294–295).^8^ But, in any event, it also commits him to rejecting Carrasco’s interpretation of her results as showing attentional effects on content in early vision—whether he might reject it on Schneider’s grounds or some other. Our arguments require no such commitment.^9^

Note also that Raftopoulos (2015), like Pylyshyn, argues that later vision is indeed cognitively penetrated. But their arguments differ. Pylyshyn, as we saw, adduces cases where cognitive states are accessed in attributing further features. While some of Raftopoulos’ arguments take this form, Raftopoulos also adverts to attentional effects not involving access to other cognitive states (Raftopoulos, 2015, pp. 283–284). Our defense of Pylyshyn would also preclude such attentional effects in later vision from counting as cognitive penetration. Cases, however, where attention is what facilitates the access of relevant cognitive states are another matter—see Raftopoulos (2015, p. 284f). Nor, more generally, would our considerations speak to the non-attention-centered arguments that Raftopoulos, like Pylyshyn, advances.

### Firestone and Scholl

Firestone and Scholl (2015, p. 36) appear to agree with our reply on Pylyshyn’s behalf when they argue that at least some covert attentional effects on perception “may be occasioned by a relevant intention or belief, but they are not sensitive to the content of that intention or belief.” (They limit their scope to “many” such effects, allowing that there may be other more “rich and nuanced” cases not covered.) But consider how they argue for their claim:

A critical commonality [with overt attention or turning off the lights], perhaps, is that the influence of attention (or eye movements) in such cases is completely independent of why you attended that way. Having the lights turned off will have the same effect on visual perception regardless of why they were turned off, including whether you turned them off intentionally or accidentally; in both cases it’s the change in the light doing the work, not the antecedent intention. And in similar fashion, attention may enhance what is seen regardless of the reasons that led you to deploy attention in that way, and even whether you attended voluntarily or via involuntary attentional capture; in both cases, it’s the change in attention doing the work, not the antecedent intention (Firestone and Scholl, 2015, pp. 35–36).

Firestone and Scholl’s main point, translated into our terms, is that it is the attentional command that does the work, regardless of what generated *it*. This, we have seen, justifies the conclusion that propositional attitudes that generate an attentional command do not satisfy Pylyshyn’s directness requirement. So, we indeed agree with Firestone and Scholl’s conclusion to this extent. But their discussion is incomplete; the directness constraint cannot by itself do all the necessary work.

Note first that Firestone and Scholl’s talk of attention’s influence on perception might suggest that, even in the covert case, we should conceive of attention as a faculty or capacity distinct from perception. But, similarly to Mole (2015), Firestone and Scholl (2016, p. 24) also write: “Our project concerns the ‘joint’ between perception and cognition, and attention unquestionably belongs on the ‘perception’ side of this joint”. The apparent tension vanishes if we import our distinction between the attentional command and the attentional effect. But doing so also helps us see that Firestone and Scholl have left undone some of the work we undertook above. At the risk of repetition, let us review how this plays out. If attention is located on the side of perception, then one might argue the generation of an attentional command from propositional attitudes is itself a *direct* effect of cognition on perception. Now we have a choice point. If we allow that the attentional command, and not just the attentional effect, is indeed located on the side of perception, then we need to argue that, though the attentional command is a representation in perception, it is not itself a perception. If we rather place the attentional command in cognition, then it is not even a candidate target of penetration and clearly the directness of its relation to the generating attitudes is irrelevant. But then we must ask about the relation between the attentional command and the resulting effect. Here directness is not the issue, but rather semantic coherence—and this constraint is absent from Firestone and Scholl’s argument.

It may seem otherwise, since they speak of sensitivity to content. But reflection on their argument makes it clear they have in mind directness, not semantic coherence. Consider a case where I in fact come to believe X by inferring it from Y, but I could have acquired belief X via hypnosis. That I *could* have acquired the belief in another way does not change the fact that I *actually* acquired it via a cognitive state that provides a reason in its favor. Similarly, as we saw, the attitudes that generate an attentional command can likewise satisfy semantic coherence (on its narrow construal) relative to the resulting perceptual state. That the same attentional command could have been otherwise generated is irrelevant, so far as semantic coherence is concerned. But these alternatives do matter for establishing indirectness, which is thus what Firestone and Scholl’s focus on the irrelevance of what caused the attentional change must be about. So, there is a distinction (between attentional commands and effects) and a further requirement for cognitive penetration (semantic coherence) that Firestone and Scholl omit.

## Two Objections

We conclude by replying to two objections. According to the first, attention should be assimilated to expectation; and, once it is, cognitively driven attentional effects, recharacterized in a Bayesian framework, seem to satisfy Pylyshyn’s requirements. According to the second, we need not stick with Pylyshyn’s characterization of cognitive penetrability in any event; and, on some other, well-motivated conceptions, cognitively driven attentional effects can indeed amount to cognitive penetration.

### Attention as Expectation

Above, we considered the objection that covert attention does not involve a distinct faculty intermediate between cognition and perception. We responded by arguing that, nonetheless, one may distinguish between an attentional command and attentional effects—adding that attentional weightings, common in computational models, are naturally construed as attentional commands. This restored a notion of attentional cause without reifying an attentional faculty.

It may be replied that this does not take sufficiently seriously the claim that attentional effects are a by-product of perceptual processing and do not involve attentional causes at all (Anderson, 2011; Vincent, 2015). The ‘by-product’ claim is often developed within a Bayesian framework that treats attentional effects as resulting from expectations (Dayan and Zemel, 1999). On the Bayesian approach, perception solves the problem of inferring the distal scene from noisy, ambiguous sensory signals by performing, or approximating, a Bayesian inference that balances the likelihood of a sensory signal, given a candidate distal cause, and the prior probability of that cause. To say, in this framework, that attentional effects result from expectations is thus to say that observed attentional effects can be accounted for in terms of the priors perception brings to bear in inferring distal causes (e.g., Rao, 2005).

If all attentional effects could be accounted for in this way, the model would require no specifically attentional parameters. For example, rather than a command to attend this much to this location, there might be an increased expectation that the target will be there. Moreover, not only would the expectation cause the attentional effect, it would do so because perception would take it into account in (quasi-) inferentially generating its output.^10^ Replacing attentional commands with expectations would thus both remove the barrier to directness and guarantee the satisfaction of semantic coherence.

This would not settle all questions concerning cognitive penetration. First, one could still ask whether the accessed expectations, particularly in cases where the effect was on *early* vision, were in fact *cognitive* states (*beliefs* about the future). Second, given recent debates concerning the intended or appropriate Marrian level of Bayesian models (e.g., Bowers and Davis, 2012a,b; Griffiths et al., 2012—and cf. Marr, 1982), one might attempt to reinstate a directness challenge elsewhere. Questions of cognitive penetrability are arguably posed at the algorithmic level, but Bayesian models are sometimes put forward as computational-level claims. If so, the question remains open whether at the algorithmic level early vision directly accesses cognitive expectations—or whether the effects of cognitive expectations are rather mediated by effects, say, on imagery (Macpherson, 2012; Block, 2016—though see Gross et al., 2014) or on visual working memory.

But, in any event, there is an antecedent problem: attention in fact dissociates from expectation (Summerfield and Egner, 2009, 2016; Summerfield and de Lange, 2014). For example, endogenous cues can direct attention even when they are uninformative about the target. Moreover, neurophysiologically attention is associated with enhanced neural response, while expectation is associated with reduced neural response (Yoshiura et al., 1999). Bayesian models without attentional parameters only handle phenomena where attention and expectation coincide; Bayesian models that attempt to address the dissociation tend to reintroduce attentional parameters (e.g., Whiteley and Sahani, 2012).^11^

It may be replied that this defense of Pylyshyn fails even if only *some* cognitively driven attentional effects can be treated as resulting from (cognitive) expectations. But the reintroduction of attentional parameters—supposing such models are accepted—argues for a natural divide among phenomena. On such a view, effects not explained attentionally are not attentional effects after all. This might seem a Pyrrhic victory, if the non-attentional expectation effects demonstrate there is cognitive penetration in any event. But this paper is not a defense of cognitive impenetrability *tout court*, only of the non-penetrability of cognitively driven attentional effects. It remains a question of course whether there *is* expectation-based cognitive penetration—recall the other issues mentioned above. But if there is, it is important to distinguish it from cognitively driven attentional effects. We want to know not just whether there is cognitive penetration, but also, if there is, the details of how it does and does not occur.

### What Should Cognitive Penetrability Be?

Finally, Pylyshyn’s characterization of cognitive penetration is not the only one. Some others also preclude attentional effects. For example, Macpherson (2012) explicitly rules out effects of spatial attention (though see Macpherson, 2015 for a change of heart):

… perceptual experience is cognitively impenetrable if it is not possible for two subjects (or one subject at different times) to have two different experiences on account of a difference in their cognitive systems which makes this difference intelligible when certain facts about the case are held fixed, namely, the nature of the [effect of the] proximal stimulus on the sensory organ, the state of the sensory organ, and the location of attentional focus of the subject. (Macpherson, 2012, p. 29)^12^

But some alternative characterizations do not preclude attentional effects. Stokes (2013, p. 650) suggests that “[a] perceptual experience E is cognitively penetrated if and only if (1) E is causally dependent upon some cognitive state C and (2) the causal link between E and C is internal and mental.” The second constraint rules out overt, but not covert attentional shifts. Wu (forthcoming) argues that cognitively driven attentional effects on perception amount to cognitive penetration by explicitly dropping Pylyshyn’s semantic coherence constraint in favor of a weaker statistical, or correlational, notion of information penetration.

Which conception of cognitive penetration should we use? Which gets the phenomenon right, or marks an important joint, or is the most fruitful? In particular, is it Pylyshyn’s? A tempting reply is that ‘cognitive penetration’ is a technical term, which Pylyshyn coined; so, how it could he fail to get it right? But someone can put their finger on something without quite articulating what matters most. Indeed, the 1999 formulation on which we have focused is itself a modification of Pylyshyn’s earlier statements. (See Stokes, 2013 for discussion and references.)

Stokes (2015) suggests we assess the candidates in terms of their consequences—especially their consequences for the questions that drive our interest in cognitive penetrability in the first place. He underscores two kinds of consequence in particular: for questions concerning cognitive architecture and for questions in epistemology. And he argues that Pylyshyn’s characterization, though it has an epistemic dimension in virtue of its requirement of semantic coherence, fails to connect with the epistemological questions. We can see this in relation more specifically to cases involving cognitively driven attention by noting, for example, their importance for issues of bias (cf. Lyons, 2015; Wu, forthcoming; Silins, 2016 surveys epistemological questions connected to cognitive penetrability). Some questions of cognitive architecture likewise seem not to turn on semantic coherence: even if valuing or desiring money affects the perceived size of coins (Bruner and Goodman, 1947—but see Landis et al., 1966), it does not provide a reason for this shift.^13^

But an alternative approach, which Stokes mentions but does not develop, would consider the consequences for various debates one at a time, instead of attempting to find a single characterization of cognitive penetrability that fits them all (cf. Siegel, 2015). Pylyshyn’s characterization (*pace* Stokes, 2013, p. 659, fn. 5) has a specific motivation, outlined above: to see whether early vision is “continuous” with cognition in virtue of early visual states standing in the same kind of relation to cognitive states that cognitive states can stand in with regard to one another. This renders of interest questions formulated using his characterization regardless of their bearing on other questions also of interest.^14^

There are many phenomena and questions of interest here. We can be pluralists about our interests. As for the label ‘cognitive penetrability,’ since discussion has proceeded in several directions, we can be pluralist about that as well, so long as we are clear. This does not mean that any characterization of ‘cognitive penetrability’ is as good as another. Some may have no interest at all. Which do will get sorted out in the light of further investigation, theoretical and empirical. We have argued that Pylyshyn’s question is of interest and that his answer, regarding attentional effects, is correct—although not for the reason he emphasizes.

## Author Contributions

The author confirms being the sole contributor of this work and approved it for publication.

## Conflict of Interest Statement

The author declares that the research was conducted in the absence of any commercial or financial relationships that could be construed as a potential conflict of interest.

## Footnotes

1 This gloss on Pylyshyn’s constraint is more demanding than others found in the literature—cf. Stokes, 2013 for discussion—but it finds support in the quoted text. If it is not evident how the content of a cognitive state might supply a reason bearing on that of a perceptual state (as opposed to vice versa), recall that, at least since Helmholtz, it has been common to think of perception as engaged in something like inference: the question then is whether (and, if so, how) cognitive states can contribute to this inference-like process. Models on which they can are discussed below.

2 Whether a cognitively driven attentional effect was brought about by pre-cueing could matter for others’ arguments. For example, as we will see, Raftopoulos (2009) puts much weight on time-course considerations. Suppose that what brings about a cognitively driven attentional effect matters to the subsequent time-course of that effect on early vision. It would then be of note if, say, attentional effects brought about by pre-cueing occurred within the window of early visual processing, while effects brought about by a decision to attend could not. The importance of such a difference would be lessened, however, if such effects need not occur “online”—that is during early visual processing—to be relevant to questions of cognitive penetrability, but could instead affect subsequent early visual processing. Raftopoulos and Zeimbekis (2015, p. 23) raise the possibility that, because “pre-cueing does not affect visual processing in a direct, online way, but just sets the initial values of certain parameters for subsequent computations,” it does not amount to cognitive penetration. But it is unclear whether computationally there is a substantive difference between directly supplying an input and fixing a parameter (and whether it matters if one happens before the other). Our discussion of semantic coherence might supply a way of cashing out this difference. But then the relevant distinction is not one particular to pre-stimulus cueing. (Note, incidentally, concerning relative timing, that what matters—if it does—would be that the input/parameter fixing from cognition occurs before sensory input from the stimulus, not that the endogenous cue that drives cognition comes before the stimulus.)

3 I say that this is his *main* argument—and elsewhere that this is the argument he *emphasizes*—because, just after providing his characterization of cognitive penetrability, he illustrates it as follows: Note that changes produced by shaping basic sensors, say by attenuating or enhancing the output of certain feature detectors (perhaps through focal attention), do not count as cognitive penetration because they do not alter the contents of perceptions in a way that is logically connected to the contents of beliefs, expectations, values, and so on, regardless of how the latter are arrived at (Pylyshyn, 1999a, p. 343). But the rest of his many remarks on attentional effects focus solely on their being prior or posterior to early vision.

4 Cf. Raftopoulos (2009) and Anderson (2011). Mole (2015) develops the point with reference to Desimone (1998) and Duncan’s (1998) biased competition model of attention. Gross (2016) critically discusses some aspects of Mole’s arguments. (For someone claiming that attention is a *cognitive* process, see Lupyan, 2015, p. 560.)

5 On some views, perception-based demonstrative reference in thought requires prior attention to the demonstratum (Campbell, 2002). The apparent circle can be avoided by denying this requirement or by distinguishing among attentional mechanisms (Wu, forthcoming).

6 I am bracketing various further nuances. For example, A might be a non-entailing reason for B, but one may not uphold B on the basis of A, not because one does not realize that A is a reason for B, but because other, overall stronger considerations lead one to deny B.

7 Of course, less schematized models can allow sub-transitions from perceptual states, weighted by attention, to further perceptual states; inputs from other sources; probabilistic states; and many other complications. And they will unpack *f*.

8 Some of Raftopoulos’ other remarks (e.g., Raftopoulos, 2009, p. 322) likewise suggest that spatial attention *does* affect perceptual content, but only in virtue of selecting what signals get (fully) processed, not in virtue of any further effect on processing. He deems such effects indirect, but it is not clear in what sense, since he allows effects of spatial attention within early vision.

9 Raftopoulos’ view that attention only facilitates processing without affecting perceptual content is in part buttressed by his view that perception delivers “rich” iconic representations. For some challenges to the evidence in favor of “rich” over “sparse” perceptual representations in vision, see Gross and Flombaum (forthcoming).

10 In effect, perception says: “This sensory signal is difficult to interpret: it could be caused by a variety of things. But the expectation of there being something red there gives me some reason to think it is more likely the signal was caused by something red than by something blue. So, let us go with that.” (We add ‘quasi’ above in deference to those who reserve the term ‘inference’ for relevant operations over *conceptual* representations—e.g., Burge, 2010.)

11 Attentional parameters are sometimes construed in terms of a different kind of expectation: an expectation concerning precision in the signal, as opposed to an expectation concerning its distal cause (e.g., Feldman and Friston, 2010—though, as it happens, they suggest the effects of endogenous cues do not involve *cognitive* states). It is suggested that, within a predictive coding framework, one can thus account for differences in neural response associated with attention and expectation concerning the stimulus. (A mechanism that increases gain is typically hypothesized, but recall Pestilli et al., 2011 and Orhan and Ma, 2015, cited above.) However, just as attention can be directed independently of expectations concerning the distal scene, it can be directed in the absence of expectations concerning stimulus precision. Attention thus dissociates from this kind of expectation as well. (An attention shift may *cause* higher precision, and in this sense it would be reasonable to expect higher precision to result. But the point is that an attention shift need not result from an *independent* expectation of precision. Thus, it cannot be construed as reason-giving.) For criticisms, consonant with our views, of predictive coding accounts of attention, see Ransom et al. (2017).

12 Macpherson (2012, pp. 43–46) is inclined, however, to allow feature-based attention. Incidentally, her formulation does not preclude *other* indirect effects: her paper defends an indirect mechanism—via mental imagery—for the cognitive penetration of color experience. See Gross et al. (2014) for discussion.

13 Semantic coherence is also not relevant for various questions concerning the causal effect of non-cognitive, but non-perceptual, states (e.g., states directing motor systems and some emotional states) on perceptual states. Of course these are not then questions of *cognitive* penetrability, but they are interesting questions nonetheless and are relevant to debates about modularity, on some characterizations.

14 Firestone and Scholl (2015, 2016) provide a different motivation for characterizations that preclude attentional effects: such effects are mainstream in perception science and fairly well-understood, whereas cognitive penetration is supposed to be a surprising, radical claim. Purveyors of the ‘all levels’ objection might respond that the goalposts have unfairly shifted: that attentional effects are bound up with perception at all levels was not so mainstream when Pylyshyn rejected the claim. We have provided an alternative Pylyshyan reply to the ‘all levels’ objection. But, in any event, perhaps Firestone and Scholl could add that, if we want to keep our questions interesting, shifting the goalposts is the right thing to do as knowledge progresses.
